# Single-pixel imaging flow cytometry for biomedical research

**DOI:** 10.1186/s41232-025-00401-5

**Published:** 2025-12-12

**Authors:** Hiroshi Kanno, Zhiying Liu, Rio Sato, Hidenori Endo, Kuniyasu Niizuma, Keisuke Goda

**Affiliations:** 1 Department of Chemistry, The University of Tokyo, Tokyo, 113-0033 Japan; 2 Department of Translational Neuroscience, Tohoku University Graduate School of Medicine, Miyagi, 980-8575 Japan; 3 Tokyo Metropolitan Kokusai High School, Tokyo, 153-0041 Japan; 4 LucasLand, Co. Ltd., Tokyo, 113-0023 Japan; 5 Department of Neurosurgery, Tohoku University Graduate School of Medicine, Miyagi, 980-8575 Japan; 6 Department of Neurosurgical Engineering, Graduate School of Biomedical Engineering, Tohoku University, Miyagi, 980-8575 Japan; 7 Institute of Technological Sciences, Wuhan University, Hubei, 430072 China; 8 Department of Bioengineering, University of California, Los Angeles, CA 90095 USA; 9 International Center for Synchrotron Radiation Innovation Smart (SRIS), Tohoku University, Miyagi, 980-8577 Japan

**Keywords:** Imaging flow cytometry, Single-pixel imaging, Cancer, Thrombosis, COVID-19, Coronary artery disease

## Abstract

High-throughput single-cell analysis and screening have become essential tools in life science research. Imaging flow cytometry, in particular, enables large-scale image-based profiling of heterogeneous cell populations, allowing statistical analysis of cellular morphology, subcellular features, and functional responses. However, its analytical capability is often limited by the use of conventional two-dimensional (2D) image sensors. In this review, we highlight recent advances in single-pixel imaging flow cytometry, which replaces 2D image sensors with single-pixel photodetectors. This approach offers advantages in sensitivity, flexibility, and speed in imaging system design and has been implemented in various optical configurations to achieve high-throughput single-cell imaging. We first introduce its key techniques, then outline representative biomedical applications, including cancer and COVID-19 research, and finally discuss current limitations and prospects for future developments. Single-pixel imaging flow cytometry is expected to serve as a versatile platform supporting both basic and translational studies in diverse biomedical applications.

## Background

High-throughput single-cell analysis and screening have become indispensable tools in life science research, as they provide insights that are masked in conventional bulk analyses. These approaches, including single-cell sequencing [1–3], mass cytometry [4, 5], and flow cytometry [6, 7], enable researchers to investigate cellular heterogeneity in complex biological systems, identify rare cell populations, and facilitate drug discovery. Specifically, single-cell sequencing and mass cytometry enable molecular-level analysis of gene expression and protein abundance at the single-cell level and have been used to identify genetic variations, mutations, and subpopulations in cancer, among other applications [8–13]. On the other hand, flow cytometry offers cell-level rapid analysis of large populations (e.g., > 10,000 cells/sec) and has been used for applications such as cell cycle analysis and cell viability assessment [14, 15]. It also enables immunolabeling-based sorting of target cells, such as fluorescence-activated cell sorting (FACS) [16–19], which is especially valuable for enriching rare populations such as multilineage-differentiating stress enduring (Muse) cells [20–23], CD34 + hematopoietic stem cells (HSCs) [24, 25], and CD4 + CD25highCD127low regulatory T cells (Tregs) [26, 27]. However, despite their strengths, these methods generally lack spatial and morphological resolution, which limits their ability to capture high-dimensional cellular morphology or dynamic processes within live cells, such as drug response.

**Fig. 1:**
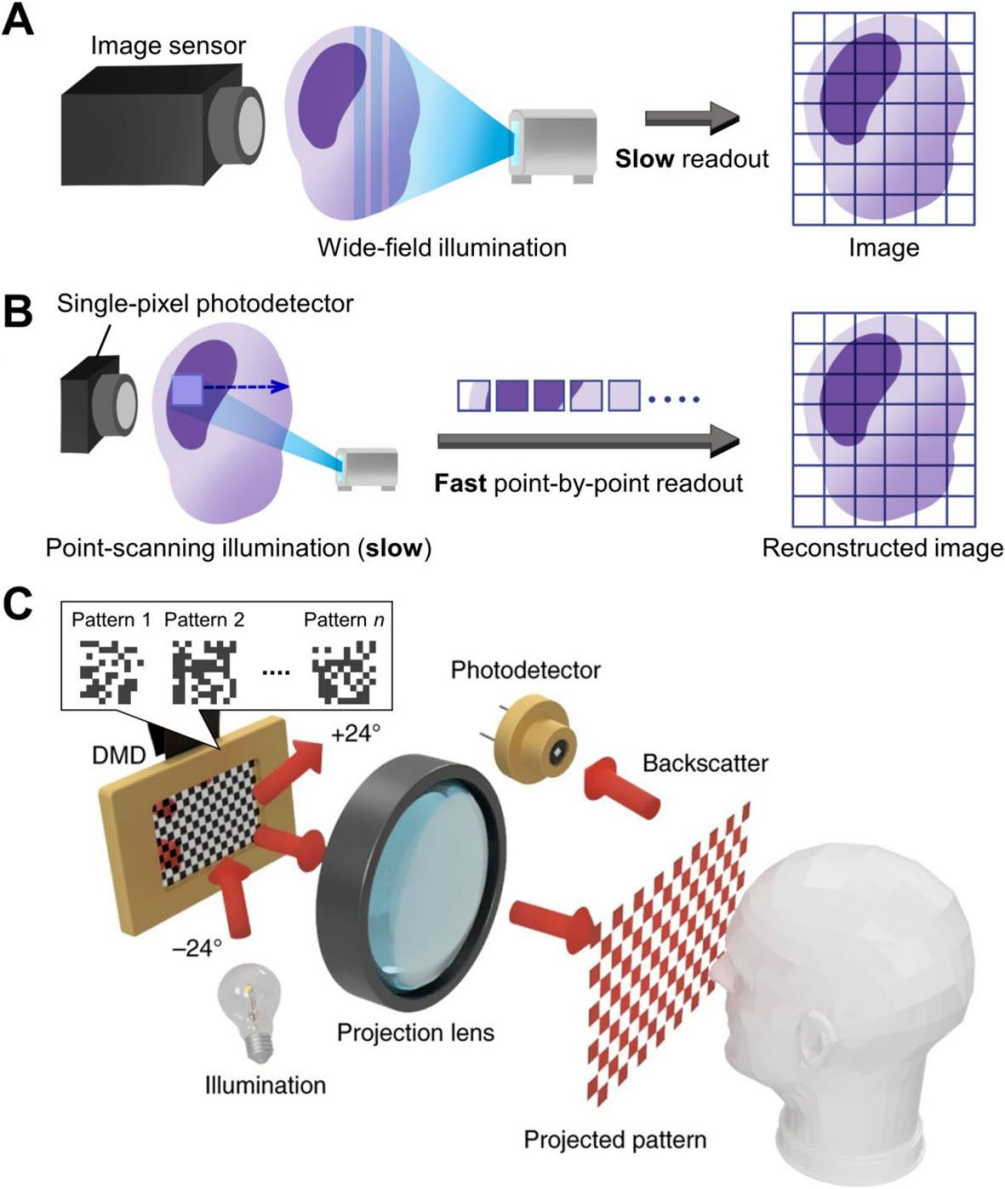
Principles of single-pixel imaging. **A** Schematic of conventional imaging using a 2D image sensor. The image readout speed of the sensor is typically slow, which limits the overall image acquisition rate. **B** Schematic of basic single-pixel imaging. Because a single-pixel photodetector lacks inherent spatial resolution, sequential beam scanning is required to reconstruct a 2D image, which typically limits the imaging speed. **C** Single-pixel imaging using pseudo-random light patterns. DMD: digital micromirror device. *n* indicates the number of illumination patterns. Adapted from Ref. [38] with permission from Springer Nature, copyright 2018

As another single-cell analysis tool, optical microscopy provides high spatial resolution for observing cellular structures, enabling detailed analysis of individual cells. Researchers can selectively visualize specific cellular components, including genomes, proteins, and organelles, and analyze their structures and functions for basic research by tagging them with appropriate fluorescent probes [28–30]. In addition, it is reliably used to examine medical samples, such as patient-resected tumors, in clinical settings to assess their diagnostic characteristics and malignancy, aiding physicians in deciding on treatment strategies [31, 32]. Furthermore, recent advances in spatial transcriptomics and automated multiplexed imaging platforms have allowed spatial mapping of dozens or more RNA or protein targets on tissue sections, expanding the capabilities of optical microscopy [33–35]. Despite these advances, analyzing a large number of cells rapidly remains challenging with standard optical microscopy due to the trade-off between spatial resolution and field of view. In this context, imaging flow cytometry, which integrates microscopy with flow cytometry, has been recently utilized to overcome the limitations of both methods. It enables large-scale image-based analysis of cellular morphology, localization of biomolecules, and cell cycle stages [36, 37]. However, its analytical capability is often limited by the use of two-dimensional (2D) image sensors, such as charge-coupled device (CCD) and complementary metal–oxide–semiconductor (CMOS) image sensors. While these sensors are easy to use and widely available for cell imaging, they generally suffer from slower image readout speeds and limited flexibility in imaging modalities compared to single-pixel photodetectors (Fig. 1A).

In this review article, we provide an overview of imaging flow cytometry based on a single-pixel imaging scheme, which replaces conventional 2D image sensors with single-pixel photodetectors, such as photomultiplier tubes (PMTs) and avalanche photodiodes (APDs), to overcome their inherent limitations. We focus on how this approach enables further improvements in sensitivity, flexibility, and speed for large-scale single-cell analysis and screening. Specifically, this review begins with the fundamental principles of single-pixel imaging, followed by its concrete implementation in the context of imaging flow cytometry. We then introduce recent applications of this technique for large-scale single-cell analysis. Finally, we discuss current limitations and future directions for development in single-pixel imaging flow cytometry and conclude with a summary of the article.

## Principles of single-pixel imaging

Single-pixel imaging has emerged as a powerful technique to overcome the limitations of conventional optical imaging methods that rely on 2D image sensors [38]. Compared with such sensors, single-pixel photodetectors are typically smaller, less expensive, mechanically simpler, faster in readout speed, and more sensitive, providing greater flexibility and scalability in imaging system design. Owing to these advantages, single-pixel imaging has been applied across diverse fields, including 3D imaging [39, 40], remote sensing [41], millimeter-wave imaging [42], time-of-flight imaging [43, 44], non-line-of-sight imaging [45, 46], high-resolution imaging [47], and high-speed imaging [48, 49]. For instance, 3D imaging can be achieved by employing multiple single-pixel photodetectors placed at different positions to collect signals from various angles and depths [39], followed by appropriate computational reconstruction. In addition, single-pixel imaging is also indispensable in spectral regions where suitable 2D image sensors are not readily available, such as the terahertz regime [50–52]. Furthermore, the fast readout speed and high gain capability of single-pixel photodetectors make them particularly advantageous for high-speed imaging, which is essential for high-throughput imaging flow cytometry.

Unlike 2D image sensors that directly record a spatial image, single-pixel photodetectors have no inherent spatial resolution, which necessitates unique illumination (or excitation in fluorescence imaging) strategies to spatially resolve a target object and computationally reconstruct its image. The simplest and most common approach is point-by-point scanning with a focused beam spot (Fig. 1B), where each target position is sequentially illuminated and detected. The image is reconstructed by mapping the temporal intensity sequence from the single-pixel photodetector into a 2D format. This straightforward approach is used when high spatial resolution and sensitivity are prioritized, as in confocal [53], multiphoton [54], and stimulated emission depletion (STED) microscopy [47]. However, the inertia of mechanical 2D scanning limits image acquisition speed, making this approach less suitable for applications requiring high temporal resolution.

An alternative approach is to illuminate the entire object with a sequence of structured light patterns, such as pseudo-random patterns. These patterns are typically generated using a light-modulating device, including a digital micromirror device (DMD) programmed with binary patterns (Fig. 1C) [38, 55]. Each illumination pattern produces a corresponding intensity signal at the single-pixel photodetector, where higher (or lower) detected intensity indicates stronger (or weaker) similarity between the pattern and the object. The image can be computationally reconstructed by correlating the known light patterns with the detected intensity sequence [56]. This approach can also take advantage of compressed sensing, which assumes that the target object is sparse, thereby reducing the number of illumination patterns required for image reconstruction [56]. However, because multiple illuminations of the same object are needed to form a single image, the overall acquisition speed is ultimately limited by how fast the light-modulating device can switch patterns. In DMD-based systems, for instance, this switching rate is typically limited to several tens of kilohertz, making it difficult to fully exploit the high temporal response (hundreds of megahertz or more) of single-pixel photodetectors. These constraints motivate the development of alternative illumination and excitation schemes specifically optimized for high-throughput imaging flow cytometry.

## Methods for single-pixel imaging flow cytometry

**Fig. 2:**
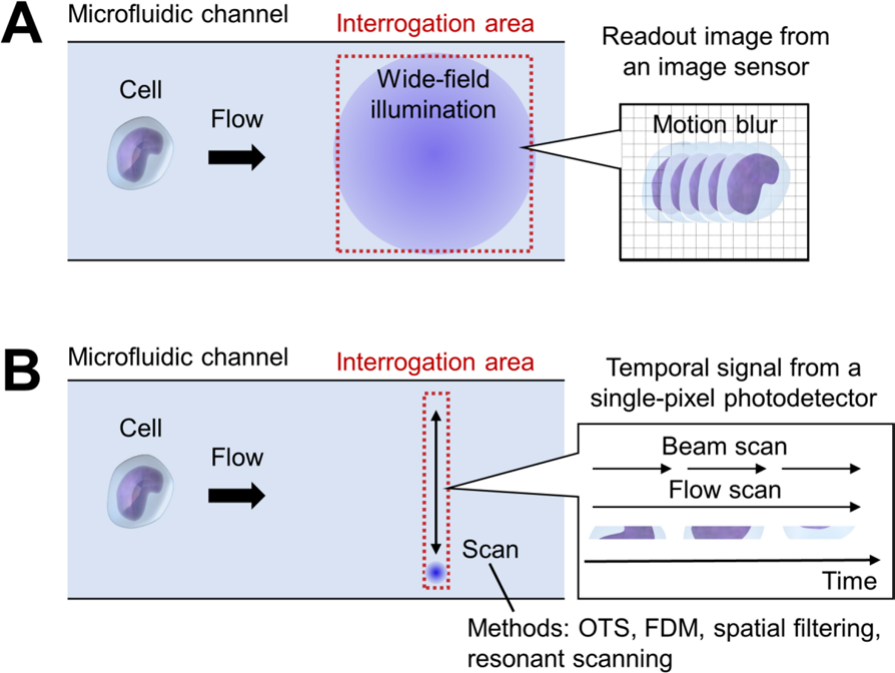
Principles of single-pixel imaging flow cytometry. **A** Schematic of conventional imaging flow cytometry using a 2D image sensor. The flow of cells is a potential source of motion blur at the image sensor. **B** Schematic of single-pixel imaging flow cytometry. The cell flow is exploited as 1D scanning of the beam spot to reconstruct a 2D image. In spatial filtering schemes, the interrogation area is typically extended along the flow direction

Single-pixel imaging flow cytometry is implemented in various configurations to enable rapid and serial image acquisition of flowing cells. In imaging flow cytometry, cells move rapidly through a microfluidic channel, potentially causing motion blur when 2D images are captured using conventional image sensors (Fig. 2A). In contrast, single-pixel imaging reconstructs 2D images through scanned illumination, allowing the linear motion of flowing cells to be advantageously utilized as 1D scanning (Fig. 2B). Consequently, only the orthogonal direction needs to be scanned to obtain 2D images. For this purpose, single-pixel imaging flow cytometry typically employs unique mechanical-scan-free illumination or detection schemes that encode spatial information of the cells onto the temporal or spectral profile of light. These approaches include optical time stretching (OTS) and frequency-division multiplexing (FDM), supporting diverse modalities such as bright-field imaging, quantitative phase imaging (QPI), and fluorescence lifetime imaging microscopy (FLIM). In the following sections, we provide a more detailed description of each method, highlighting their principles, optical configurations, and performance characteristics. Table 1 summarizes the specifications of different single-pixel imaging flow cytometry techniques.

**Table 1:** Comparison of single-pixel imaging flow cytometry techniques

Imaging flow cytometry scheme	Line rate (lines/sec)^*1^	Spatial resolution^*2^	Feature	Limitation/Requirement	Ref
OTS imaging	10^7^–10^9^	< 1 μm	High-speed imaging capability; compatibility with QPI	Requirement of a femtosecond pulse laser and a high-speed digitizer	[57–62]
FACED imaging	10^7^–10^8^	< 1 μm	High-speed imaging capability; compatibility with QPI and fluorescence imaging	Requirement of a femtosecond pulse laser and a high-speed digitizer	[63–66]
SLIDE imaging	10^5^	< 1 μm	Capability for two-photon fluorescence imaging and FLIM	Requirement of high-speed light modulating devices and a high-speed digitizer	[67]
FDM imaging	10^5^–10^6^	< 1 μm	Capability for fluorescence imaging and FLIM	Signal-to-noise ratio (SNR) reduction due to cumulative shot noise from multiple beam spots	[68–72]
Imaging with spatial filtering	10^5^	1–2 μm	Capability for 3D fluorescence and side-scattered imaging with simple illumination optics	Potential photobleaching due to inefficient light-sheet illumination; limited cell throughput due to an extended interrogation area	[73]
SRS imaging	10^4^	< 2 μm^*3^	High chemical specificity	Limited imaging speed due to weak SRS signals and the use of resonant scanning	[74]

^*^^1^ A line rate of ~ 4 × 10^6^ lines/sec reaches an event rate of ~ 10,000 cells/sec (eukaryotic cells) [72], except for imaging with spatial filtering. This configuration requires an extended illumination region along the flow direction; therefore, the cell concentration should be kept low to avoid simultaneous illumination of multiple cells, resulting in lower event rates

^*^^2^ Spatial resolution depends on experimental parameters, such as the laser wavelength and the numerical aperture of the objective lens; therefore, it does not necessarily reflect the inherent characteristics of each imaging flow cytometry technique

^*^^3^ Reported in [75], where a photodiode array was used

### Optical time-stretch imaging flow cytometry and related approaches

**Fig. 3:**
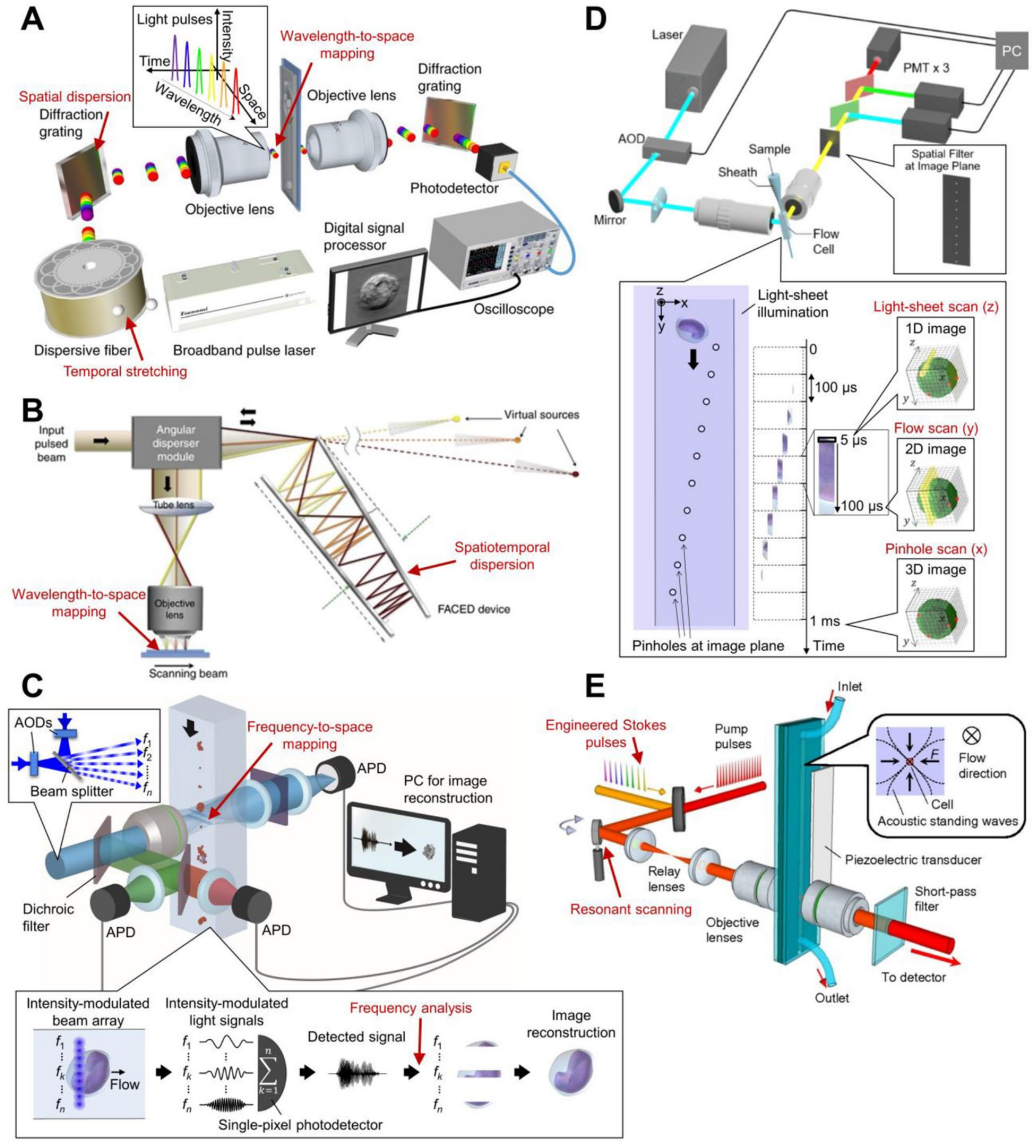
Methods for single-pixel imaging flow cytometry. **A** Schematic of the OTS imaging flow cytometer, which typically consists of a broadband pulse laser, a dispersive fiber, diffraction gratings, and a single-pixel photodetector. Owing to temporal stretching and spatial dispersion, different wavelength components of the laser pulses are mapped to different spatial and temporal positions, as shown in the inset, enabling rapid 1D scanning. Adapted from Lei C. et al., Nature Protocols, 2018 [57]. **B** Schematic of the illumination optics of the FACED imaging flow cytometer. Two opposing mirrors generate spatiotemporal dispersion of the laser pulses, as if they were emitted from multiple virtual sources, which are used for rapid 1D scanning in the same manner as in OTS imaging. Adapted from Ref. [65], licensed under CC BY 4.0. **C** Schematic of the FDM imaging flow cytometer. As shown in the lower inset, each beam spot is modulated at a distinct frequency (e.g., *f*_1_, *f*_*k*_, and *f*_*n*_) and mapped to a corresponding spatial position. The single-pixel photodetector detects a combined optical signal containing all these frequency components, which can be separated through frequency analysis to reconstruct a 2D image. Adapted from Ref. [76], licensed under CC BY 4.0. **D** Schematic of the imaging flow cytometer with a pinhole-array spatial filter. The light sheet is scanned along the z-axis to illuminate flowing cells, while fluorescence and side-scattered signals are detected at each spatial point through the corresponding pinhole. The three axes of the reconstructed 3D image correspond to light-sheet scanning, cell flow, and the alignment of the filter aperture, with respective scanning times of 5 μs, 100 μs, and 1 ms (for a flow speed of 0.2 m/s). Adapted from Ref. [73], under the Optica Publishing Group Open Access Publishing Agreement (OAPA). **E** Schematic of the SRS imaging flow cytometer. The pulses are scanned along the axis orthogonal to the flow direction using a resonant scanner. Adapted from Suzuki Y. et al., PNAS, 2019 [74]

OTS imaging flow cytometry captures images of flowing cells by illuminating them with broadband laser pulses that are both temporally and spatially dispersed [57]. As illustrated in Fig. 3A, the laser pulses are first temporally stretched as they propagate through a dispersive fiber due to chromatic dispersion. This stretching process is essential because ultrashort femtosecond pulses (~ 10^–15^ s) from a titanium-sapphire laser are orders of magnitude shorter than the temporal resolution of typical single-pixel photodetectors, such as PMTs and APDs, which have temporal resolutions of only a few hundred picoseconds (~ 10^–10^ s). By extending the pulse duration to the nanosecond range (~ 10^–9^–10^–8^ s), the dispersed light can be directly detected and digitized using standard commercial instruments. The temporally stretched light pulses are then spatially dispersed by a diffraction grating and focused by an objective lens onto the flowing cells. In this configuration, different wavelength components of each pulse illuminate different positions sequentially along the axis orthogonal to the flow. As the cell flows through the microfluidic channel, each successive pulse effectively scans a different line of the cell. The transmitted pulses are collected and converted back to their original pulse form by another diffraction grating before being detected by a single-pixel photodetector. Finally, the detected temporal intensity signals are digitized and straightforwardly reconstructed into 2D images. Owing to this mechanical-scanning-free, all-optical line-scanning scheme, OTS imaging flow cytometry can achieve line-scan rates in the megahertz to gigahertz range, corresponding to 10,000–1,000,000 cell image acquisitions per second [58, 59].

OTS imaging flow cytometry also supports modalities other than bright-field imaging. Specifically, it can be configured for QPI by introducing a reference beam to interfere with the transmitted light [60–62]. This enables label-free visualization of optical path difference maps, which can be used to quantitatively estimate cell dry mass [77]. More recently, a virtual OTS-QPI approach has been demonstrated using deep learning, which eliminates the need for complex interferometric setups and avoids phase unwrapping errors [78]. In addition, a conceptually related single-pixel imaging technique, known as spectro-temporal laser imaging by diffracted excitation (SLIDE), has been developed for two-photon FLIM [67]. In SLIDE, each spatial point of a flowing cell is excited by an individual pulse with a distinct wavelength that is completely separated in time from the others, allowing sequential recording of fluorescence intensity decay without temporal overlap. This enables fluorescence lifetime imaging of cells flowing at speeds of up to 0.2 m/s.

Another similar concept of OTS imaging is realized in a different implementation called free-space angular-chirp-enhanced delay (FACED), which employs two opposing mirrors positioned with a slight tilt to achieve simultaneous temporal and spatial dispersion [63–65]. As shown in Fig. 3B, when each light pulse converges and enters the mirror pair, the light components of the pulse follow distinct optical paths determined by their incident angles while being reflected. This results in the generation of temporally and spatially dispersed light pulses at the point of ejection from the mirror pair. This is equivalent to light pulses being emitted from multiple point optical sources placed at different positions (Fig. 3B). Converging the light pulses to the entrance of the mirror pair can be accomplished by using either a diffraction grating or a cylindrical lens before the mirror pair [65]. The former approach is equivalent to OTS imaging in that the optical spectrum corresponds one-to-one with spatial position, whereas the latter is not limited to OTS imaging. FACED also supports fluorescence imaging and QPI [63, 64, 66].

### Frequency-division-multiplexed imaging flow cytometry

FDM imaging flow cytometry enables high-speed image acquisition of flowing cells using a continuous-wave laser [68–71, 79–83]. The key idea is to encode spatial information of the cells into different modulation frequencies of light, which is an optical adaptation of a signal multiplexing technique widely used in telecommunications [84–88]. In this approach, flowing cells are illuminated with an intensity-modulated beam array, in which each beam spot is modulated at a distinct frequency (Fig. 3C). As the cells flow through the illumination region, each point on the cell is probed by a specific modulation frequency. The transmitted or fluorescence light signals from all illuminated spots are then simultaneously detected by a single-pixel photodetector and later separated into individual spatial channels through digital frequency analysis, including fast Fourier transformation.

In a typical FDM imaging flow cytometer, the intensity-modulated beam array is generated by an interferometer incorporating acousto-optic devices, such as acousto-optic deflectors (AODs). When driven by multi-tone signals, the AODs deflect the laser beam into multiple directions while inducing frequency shifts, as shown in the upper inset of Fig. 3C. The interference of these beams generates a spatially distributed pattern of intensity modulation, in which each position along the axis perpendicular to the cell flow corresponds to a unique modulation frequency. Consequently, FDM imaging flow cytometry achieves line-scan rates in the megahertz range, allowing image acquisition of around 10,000 cells per second [72, 89]. Additionally, FDM imaging flow cytometry has also been recently extended to FLIM by using a pair of beam arrays [72, 90]. In this configuration, the phase shift between the excitation modulation and the detected fluorescence signal corresponds to fluorescence lifetime, enabling lifetime-resolved imaging of flowing cells at 10,000 cells per second without sacrificing imaging speed or signal-to-noise ratio. Furthermore, integration of FDM imaging flow cytometry with a real-time sorting mechanism led to the development of the first intelligent image-activated cell sorter (iIACS) [91–94].

### Imaging flow cytometry with spatial filtering

Imaging flow cytometry with spatial filtering represents another class of single-pixel imaging approaches that rely on specialized detection rather than structured illumination. Unlike OTS and FDM imaging flow cytometry, these approaches use wide-field illumination and instead extract spatial information of flowing cells using custom-designed spatial filters placed at an intermediate image plane (Fig. 3D) [73, 95–97]. This spatial filter selectively transmits optical signals from specific regions of the flowing cells while blocking unwanted background light. Specifically, one implementation incorporated a stair-step pattern of rectangular apertures into a conventional flow cytometer, effectively converting it to an imaging flow cytometer [95]. Another approach combined a spatial filter with multiple spot apertures and light-sheet illumination, enabling 3D fluorescence and side-scattering imaging of flowing cells [73, 96, 97]. As shown in Fig. 3D, while the cell flows through the light sheet, spatial filtering allows sequential detection of optical signals from individual volume elements (voxels). The recorded temporal signals from the single-pixel photodetector can then be aligned in sequence to reconstruct the 3D image. More recently, a related approach demonstrated spatially resolved excitation using a linear spot array generated by a diffractive optical element [98]. This technique eliminates the need for spatial filters while still employing pinhole arrays to enhance imaging quality.

### Imaging flow cytometry based on stimulated Raman scattering

Single-pixel imaging flow cytometry based on stimulated Raman scattering (SRS) enables label-free chemical imaging of single cells by probing intrinsic vibrational signatures of biomolecules [74]. In SRS, two synchronized laser pulses, called the pump and Stokes pulses, interact with molecules in the cell. When their wavelength difference matches a specific molecular vibration, the intensity of the pump beam changes slightly, allowing much easier detection than weak spontaneous Raman signals. In this SRS-based imaging flow cytometry, synchronized pump–stokes laser pulse pairs are used. The Stokes pulses contain four wavelength components that alternate every two pulses (corresponding to 52.5 ns) (Fig. 3E). The combined beams are focused into a microfluidic channel and scanned perpendicular to the flow direction using a 24-kHz resonant scanner. The transmitted pump pulses exhibit small intensity changes due to SRS, which are detected by a single-pixel photodetector and demodulated using a lock-in amplifier. By tuning the pump–Stokes wavelength difference, images corresponding to four distinct Raman shifts can be obtained and reconstructed into 2D images. Although the demonstrated flow speed (2 cm/s) is one to three orders of magnitude lower than that achieved in bright-field and fluorescence-based single-pixel imaging flow cytometry, mainly due to weak SRS signals, this approach provides superior molecular specificity.

## Applications of single-pixel imaging flow cytometry

The high-speed imaging capability of single-pixel imaging flow cytometry enables large-scale single-cell analysis based on extensive image datasets. Morphological features extracted from these datasets provide information on cell populations that cannot be obtained by conventional non-imaging flow cytometry. Moreover, when combined with machine learning techniques, including deep learning, which can capture and learn morphological patterns of cell populations, these systems allow efficient and comprehensive analysis, opening opportunities for applications beyond the reach of conventional methods. In the following sections, we highlight representative applications of single-pixel imaging flow cytometry.

### Cancer

Cancer often exhibits marked phenotypic inter- and intra-tumor heterogeneity, resulting from multiple interconnected mechanisms, including genomic instability and plasticity of cancer cells, as well as the presence of cancer stem cells [9, 99–101]. Even in cultured cell lines, heterogeneity can be observed in morphology and functional capabilities, which can arise from differences in cell cycle states and other factors. In this context, single-pixel imaging flow cytometry provides a powerful approach for comprehensively characterizing heterogeneous cancer cell populations.

**Fig. 4:**
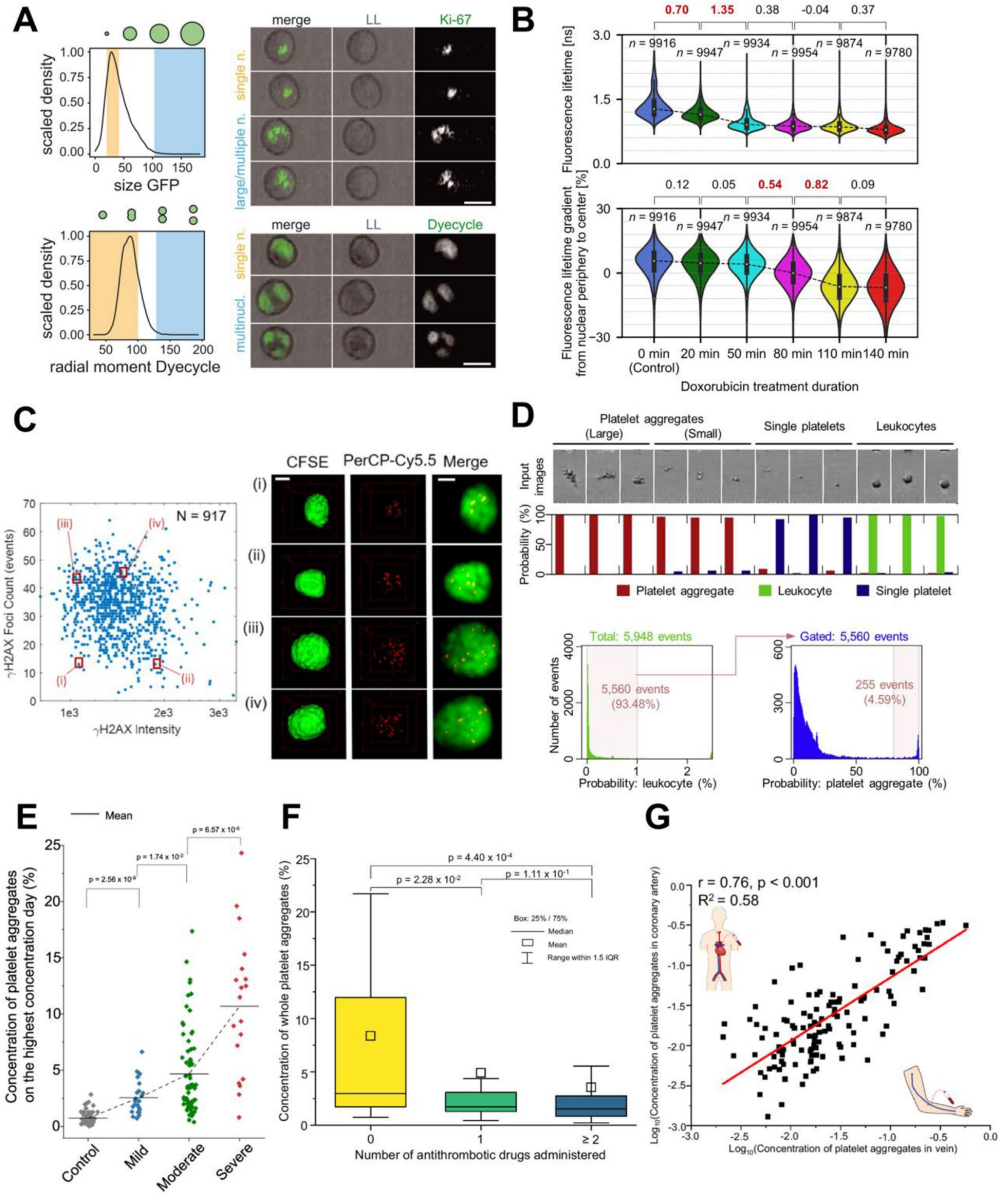
Applications of single-pixel imaging flow cytometry. **A** Histograms of HeLa cells expressing eGFP-Ki-67 (top) and stained with DyeCycle Green (bottom), with representative images corresponding to the colored regions. The orange area represents frequent events, while the blue area represents rare events. LL: light loss (bright-field images). Scale bars: 20 μm. Adapted from Ref. [102] with permission from AAAS, copyright 2022. **B** Temporal changes in fluorescence lifetime of Jurkat cells treated with doxorubicin and stained with SYTO16. Numbers above violin plots indicate effect sizes (Cohen’s *d*), showing the magnitude of difference between two cell populations. The upper panel shows distributions of average fluorescence lifetime, while the lower panel shows distributions of intranuclear fluorescence lifetime variation (i.e., gradient within the nucleus). Adapted from Ref. [72], licensed under CC BY 4.0. **C** Scatterplot of γH2AX intensity versus foci count, along with representative fluorescence images from the marked regions (i)–(iv). CFSE: carboxyfluorescein dye, PerCP-Cy5.5: DNA damage antibody-conjugated dye. Scale bar: 5 μm. Adapted from Ref. [73], under the Optica Publishing Group OAPA. **D** Probability distributions calculated by a CNN for bright-field images (top) and gating strategy for sorting platelet aggregates with high specificity using iIACS (bottom). Adapted from Ref. [91] with permission from Elsevier, copyright 2018. **E** Comparison of platelet aggregate concentrations across COVID-19 severity groups (mild: *n* = 23, moderate: *n* = 68, severe: *n* = 19). Adapted from Ref. [76], licensed under CC BY 4.0. **F** Comparison of platelet aggregate concentrations among CAD patients receiving different antithrombotic therapies (no drug administration: *n* = 28, one drug administration: *n* = 68, ≥ two drug administration: *n* = 111). Reused from Ref. [103], licensed under CC BY-NC-ND 4.0. **G** Correlation between platelet aggregate concentrations in venous and coronary artery blood of CAD patients (*n* = 129). Reused from Ref. [103], licensed under CC BY-NC-ND 4.0

An FDM imaging flow cytometry study, for example, characterized heterogeneity in HeLa cervical cancer cells, including differences in the size and number of nucleoli and nuclei as well as overall cell shape, serving as a proof-of-concept for the system (Fig. 4A) [102]. This platform also enabled sorting of cells by mitotic stage (e.g., metaphase, anaphase, and telophase) without using chemical blockers to arrest cells at specific points in the cell cycle. Similarly, label-free tracking of cell cycle stages of MDA-MB231 and MCF-7 breast cancer cells was achieved using FACED-QPI [64]. In addition, SRS imaging flow cytometry demonstrated that cancer cells artificially spiked into blood can be identified without fluorescent labels, showing their potential for label-free detection of circulating tumor cells (CTCs), which are precursors to cancer metastasis [74].

Glioblastoma is the most aggressive primary brain cancer, with a median overall survival of approximately 12–15 months despite standard therapy [104, 105]. Therefore, substantial efforts are needed to improve patient outcomes. To this end, high-throughput FLIM-based flow cytometry was applied to investigate cellular heterogeneity within a rat glioma [72]. Since fluorescence lifetime is independent of unwanted variations in fluorescence intensity, FLIM flow cytometry can offer greater robustness and precision than intensity-based approaches [90]. In this study, GS-9L rat glioma cells were implanted into rat brains, and the resulting tumors were dissociated into single cells, which were then stained with SYTO16, a nuclear-staining dye, and analyzed by FLIM flow cytometry. The study revealed intratumor heterogeneity in the fluorescence lifetime of SYTO16 and its relationship with cellular and nuclear morphological features [72]. Although precise cell type identification was not achieved, fluorescence lifetime emerged as a promising metric for studying intratumor heterogeneity.

Investigating how cancer cells respond to drugs or radiation is crucial for identifying new therapeutic strategies. To explore this, the drug responses of MCF-7 cells were investigated by label-free OTS imaging flow cytometry [106]. Classification between drug-treated and untreated cells was achieved with 92% accuracy using machine learning, demonstrating the potential of label-free drug screening. Additionally, an FDM imaging flow cytometer with sorting capability was used to study drug-induced organelle responses and pathway-specific nuclear translocation of intracellular proteins, which can be used for pooled CRISPR screens to identify genes regulating cellular signaling pathways [102]. Moreover, FLIM flow cytometry demonstrated the time-course drug responses of Jurkat leukemia cell nuclei, as reflected in the intranuclear fluorescence lifetime distribution [72]. In this study, the overall fluorescence lifetime of the cells significantly changed within 50 min after the initiation of drug treatment, whereas the intranuclear distribution of fluorescence lifetime exhibited pronounced changes between 50 and 110 min (Fig. 4B). These results suggest that the drug initially accumulated in the cell nuclei, altering the overall fluorescence lifetime, followed by nuclear structural changes affecting the intranuclear fluorescence lifetime distribution. This provided insights into cellular dynamics that could not be captured by conventional fluorescence intensity–based analysis, demonstrating the high analytical capability of FLIM-based imaging flow cytometry. Furthermore, single-pixel 3D imaging flow cytometry was used to evaluate radiation-induced DNA damage in CMK3 glioblastoma cells by directly counting γH2AX foci formed at DNA double-stranded breaks [73]. DNA damage was visualized through immunolabeling of γH2AX [107] and quantified by counting the number of fluorescent foci within each nucleus (Fig. 4C). The analysis revealed that the number of foci did not correlate with the overall fluorescence intensity, highlighting a key limitation of conventional flow cytometry for this purpose and underscoring the advantage of 3D imaging for precise DNA damage quantification. Collectively, these studies demonstrate that single-pixel imaging flow cytometry is a powerful approach for analyzing cancer cell heterogeneity and responses to drugs and radiation with potential applications in therapeutic development.

### Thrombosis

As platelet activation plays a key role in thrombosis, accurate monitoring and evaluation of platelet activity are essential for a better understanding of thrombotic events. However, microthrombi composed of platelets with or without other blood cells (i.e., platelet aggregates) are small and heterogeneous in size, morphology, and composition, making their detection, characterization, and classification traditionally challenging. In this context, iIACS enabled the rapid acquisition of bright-field images of single platelets, leukocytes, and platelet aggregates and performed real-time cell sorting with 99% specificity using a deep convolutional neural network (CNN) (Fig. 4D) [91]. The sorted platelets can subsequently be used for downstream analyses, such as transcriptomics. In another study, platelet aggregates induced by different agonists (e.g., collagen) were analyzed using a CNN trained on more than 60,000 bright-field images obtained by OTS imaging. The aggregates were successfully classified according to the type of agonist, based on subtle morphological features recognized by the CNN [108]. Given that the type of agonist is associated with different forms of thrombosis, these findings suggest the potential of a new diagnostic approach to thrombosis using patient-derived platelet aggregate images [109]. In a further study, platelet aggregates from COVID-19 and non-COVID-19 thrombosis patients were classified using FDM imaging flow cytometry and machine learning [110], while OTS imaging flow cytometry was applied to investigate stenosis-induced platelet aggregation and assess the efficacy of antiplatelet drugs [111, 112].

### COVID-19

COVID-19 first emerged in late 2019 and later became a global pandemic, with clinical manifestations including respiratory distress and a high incidence of thrombosis, particularly microvascular thrombosis [113–116]. However, conventional laboratory tests, such as light transmission aggregometry [117] and D-dimer testing [118], were insufficient to fully capture the dynamics of platelet activation and aggregation underlying these thrombotic events. To overcome this limitation, FDM imaging flow cytometry was applied to directly observe and statistically analyze circulating platelet aggregates in more than 100 hospitalized COVID-19 patients [76]. This study revealed a significant increase in the concentration of circulating platelet aggregates in the majority of patients (87.3%), thereby directly visualizing a hallmark of microthrombus formation in COVID-19. Importantly, the concentration of platelet aggregates (defined as the ratio of platelet aggregate images to all captured platelet-related images in imaging flow cytometry) showed a strong correlation with clinical parameters, such as disease severity (Fig. 4E). Furthermore, longitudinal monitoring over several months revealed that temporal changes in platelet aggregate concentrations differed according to disease severity, with distinct patterns observed between mild, moderate, and severe cases. These findings demonstrate the potential of single-pixel imaging flow cytometry to provide quantitative and mechanistic insights into COVID-19-related platelet activation, underscoring its value as a diagnostic and prognostic tool.

The same approach was extended to investigate the effects of mRNA vaccination for COVID-19 on platelet activation [119]. While mRNA vaccines developed by Pfizer-BioNTech and Moderna demonstrated strong efficacy in preventing infection and in reducing the risk of severe disease [120, 121], rare side effects, such as myocardial infarction and thrombotic events, raised concerns about their potential impact on platelet function [122]. Using large-scale image-based platelet aggregate profiling, concentrations of platelet aggregates were monitored for over 11 months in healthy volunteers who received Pfizer-BioNTech (BNT162b2) vaccinations. This study did not reveal persistent vaccination-related increases in platelet aggregate concentrations, providing supportive evidence for vaccine safety.

### Coronary artery disease

Coronary artery disease (CAD) is one of the leading causes of death globally and poses a significant challenge to global health [123]. Since platelet aggregation and resulting thrombus formation play a critical role in CAD [116], antiplatelet therapy is a cornerstone of its management [124]. However, conventional platelet function tests provide only indirect information on platelet function, limiting the ability to optimize therapy and balance the risk of thrombosis versus bleeding. To better understand CAD and evaluate antiplatelet therapy, image-based single-cell profiling using FDM imaging flow cytometry was applied [103]. In this study, platelets and platelet aggregates in blood samples collected from 207 CAD patients were imaged and statistically analyzed using deep learning-based phenotypic classification [117]. The results showed that CAD patients had higher levels of platelet aggregates compared with healthy subjects, with acute coronary syndrome patients showing higher levels than chronic coronary syndrome patients. In addition, CAD patients undergoing antiplatelet therapy demonstrated reduced platelet aggregate concentrations compared with those not receiving the therapy (Fig. 4F). Furthermore, platelet aggregation levels in coronary blood were found to correlate with those in venous blood (Fig. 4G), suggesting that venous sampling could serve as a practical surrogate marker for evaluating antiplatelet therapy and monitoring disease status in CAD patients. These findings highlight the potential of single-pixel imaging flow cytometry to provide mechanistic insights into platelet biology in CAD and to serve as a precision tool for monitoring and optimizing antiplatelet therapy [125].

## Current limitations and outlook

Although some preliminary studies have explored patient-derived samples, most applications of single-pixel imaging flow cytometry still focus on cultured cells. This limitation partly arises from the inherent complexity of single-pixel imaging flow cytometry, which relies on engineered illumination or detection schemes, sophisticated optical and electronic components, and precise flow control systems. Setting up and maintaining these systems requires specialized expertise and considerable effort. These requirements make the technique less accessible than conventional flow cytometers and imaging flow cytometers. Additionally, despite the high imaging speed of single-pixel imaging flow cytometry (e.g., > 10,000 cells/sec), analyzing the resulting large image dataset remains a bottleneck. Image data typically need to be processed offline for comprehensive cell population analysis, which limits routine clinical applications such as blood testing. Moreover, despite its promising demonstrations, a “killer application” that defines a clinical, industrial, or scientific use has not yet been clearly identified.

In the future, broader clinical applications are expected, including the analysis of patient-derived samples and potential use in rapid intraoperative diagnosis [126]. To achieve this, the development of more user-friendly instruments with improved stability, reproducibility, and compactness will be essential, for example, through the use of metasurface technology [127]. In addition, artificial intelligence (AI)-based analysis techniques are expected to be increasingly integrated into single-pixel imaging flow cytometry [128–131]. Another promising direction is its use for rare therapeutic cell populations. In particular, if clinically validated and safe subsets, such as Muse cells [132], HSCs [133], and Tregs [134, 135], could be classified and subsequently isolated in a label-free and high-throughput manner, this technology would provide a powerful platform for both basic research and regenerative medicine applications.

Extending single-pixel imaging flow cytometry to in vivo applications could represent a promising future direction. Single-pixel imaging is inherently compatible with in vivo use because it relies on point illumination, which is fundamental to multi-photon imaging modalities (i.e., multi-photon fluorescence and multi-harmonic generation) capable of high penetration depth [54]. In fact, FACED has already been applied to observe cerebral blood flow in awake mice via two-photon fluorescence imaging of red blood cells [136, 137]. If in vivo detection and classification of rare cells, such as platelet aggregates, leukocytes, and CTCs, can be realized, it would open unprecedented opportunities for minimally invasive, real-time monitoring of dynamic cellular processes and pathological conditions.

A potential hurdle, however, is that the flow velocity in superficial vessels available for observation may be insufficient to rapidly accumulate enough cells for statistical analysis. For example, the fastest flow speed reported in the previous study on mouse cerebral vessels was 0.049 m/s [136], which is one to two orders of magnitude slower than typical in vitro imaging flow cytometry. While abundant cells such as red blood cells may be observed within a short period, relatively rare cells such as leukocytes would be difficult to capture in sufficient numbers. Potential strategies to conceptually address this limitation include expanding the field of view to increase the number of cells imaged per unit time or developing wearable devices that enable prolonged observation without imposing additional stress on living subjects. Photodamage is another critical factor that must be carefully considered in the design of in vivo systems.

Beyond image-based cell population analysis, single-pixel imaging flow cytometry is expected to be increasingly integrated with downstream single-cell analyses. For example, iIACS allows real-time image-based isolation of cells for subsequent multi-omics studies [75, 91–94]. While not a sorting method, a cell placement robot combined with an imaging flow cytometer enables mapping of single-cell images to cell positions for subsequent sequencing [97]. These approaches are likely to see broader adoption and will help researchers link morphological and functional features of cells with molecular profiles, thereby expanding the scope of high-throughput single-cell analyses. Furthermore, such techniques could be applied to immunotherapy research; for instance, identifying and isolating rare T cell–cancer cell complexes could facilitate T cell receptor and neoantigen analyses, ultimately supporting the development and optimization of chimeric antigen receptor-T cell (CAR-T) therapies [97, 138].

## Conclusions

Single-pixel imaging flow cytometry has emerged as a powerful alternative to conventional image sensor-based approaches, offering unique advantages in sensitivity, flexibility, and speed. Through implementations such as OTS, FDM, and FACED, this framework has enabled high-throughput single-cell imaging at unprecedented speeds. In addition, single-pixel imaging flow cytometry has been successfully applied to a range of biomedical applications, including cancer and thrombosis research. Furthermore, recent developments such as iIACS highlight the potential to bridge the gap between image-based analyses and downstream molecular profiling, while the compatibility of single-pixel imaging schemes with multiphoton imaging modalities suggests opportunities for in vivo applications. Continued advances in optical design, computational reconstruction, and data-driven analysis are expected to further establish single-pixel imaging flow cytometry as a versatile platform for basic and translational research.

## Data Availability

There are no new data presented, as this is a review paper.

## Abbreviations

FACS: Fluorescence-activated cell sorting/ sorter
Muse: Multilineage-differentiating stress enduring
HSC: Hematopoietic stem cell
Treg: Regulatory T cell
CCD: Charge-coupled device
CMOS: Complementary metal oxide semiconductor
PMT: Photomultiplier tube
APD: Avalanche photodiode
STED: Stimulated emission depletion
DMD: Digital micromirror device
OTS: Optical time stretch
FDM: Frequency-division multiplexing
QPI: Quantitative phase imaging
FLIM: Fluorescence lifetime imaging microscopy
SLIDE: Spectro-temporal laser imaging by diffracted excitation
FACED: Free-space angular-chirp-enhanced delay
AOD: Acousto-optic deflector
iIACS: Intelligent image-activated cell sorting/ sorter
SRS: Stimulated Raman scattering
SNR: Signal-to-noise ratio
CTC: Circulating tumor cell
CRISPR: Clustered regularly interspaced short palindromic repeats
CNN: Convolutional neural network
CAD: Coronary artery disease
AI: Artificial intelligence
CAR-T: Chimeric antigen receptor-T cell
